# Subjective Ratings of *Beauty* and *Aesthetics*: Correlations With Statistical Image Properties in Western Oil Paintings

**DOI:** 10.1177/2041669517715474

**Published:** 2017-06-28

**Authors:** Gregor U. Hayn-Leichsenring, Thomas Lehmann, Christoph Redies

**Affiliations:** DFG Research Unit Person Perception, Psychology of Beauty Group, Institute of Anatomy I, Jena University Hospital, Germany; Institute of Medical Statistics, Computer Sciences and Documentation, Jena University Hospital, Germany; Experimental Aesthetics Group, Institute of Anatomy I, Jena University Hospital, Germany

**Keywords:** low-level properties, beauty, aesthetics, oil paintings, clustering

## Abstract

For centuries, oil paintings have been a major segment of the visual arts. The JenAesthetics data set consists of a large number of high-quality images of oil paintings of Western provenance from different art periods. With this database, we studied the relationship between objective image measures and subjective evaluations of the images, especially evaluations on *aesthetics* (defined as artistic value) and *beauty* (defined as individual liking). The objective measures represented low-level statistical image properties that have been associated with aesthetic value in previous research. Subjective rating scores on *aesthetics* and *beauty* correlated not only with each other but also with different combinations of the objective measures. Furthermore, we found that paintings from different art periods vary with regard to the objective measures, that is, they exhibit specific patterns of statistical image properties. In addition, clusters of participants preferred different combinations of these properties. In conclusion, the results of the present study provide evidence that statistical image properties vary between art periods and subject matters and, in addition, they correlate with the subjective evaluation of paintings by the participants.

## Introduction

In his book *Vorschule der Ästhetik*, Gustav Theodor Fechner (1876) laid the foundations for a new scientific discipline, that is, the systematic search for stimulus properties that are associated with beauty (experimental aesthetics). He was one of the first to directly measure such properties in aesthetic stimuli. Nowadays, a large number of firmly established empirical methods are applied in this field. A particular focus has been on statistical image properties (SIPs) that are relevant to visual perception in humans. For many years, researchers have explored whether SIPs provide objective criteria to assess the aesthetic quality of artworks and photographs. Their goal was (and is) to identify universal features that are positively correlated with *beauty* and *aesthetic* experience. Until now, no such universals have been found. The main reason for this might be that experiences of beauty are—at least partly—domain specific (Marković, 2014). For example, the concept that the Golden Section—as proposed by Gustav Theodor Fechner—is universally perceived as beautiful across domains has been questioned and eventually rebutted (McManus, 1980; Russell, 2000). Over the past few years, more sophisticated analysis methods have been used to search for properties related to beauty in a specific domain, namely visual art (Graham & Redies, 2010; Hoenig, 2005). In the field of computational aesthetics, computer-assisted algorithms were used to extract statistical features that characterize aesthetic images (Amirshahi, Koch, Denzler, & Redies, 2012; Datta, Joshi, Li, & Wang, 2006; Graham & Field, 2007; Redies, Hasenstein, & Denzler, 2007). This approach has also been employed to predict emotional responses to paintings (Yanulevskaya et al., 2012) and to categorize painting styles (Wallraven et al., 2009).

Large subsets of Western and East Asian visual artworks share the property of a nearly scale-invariant (fractal-like) Fourier spectrum (Alvarez-Ramirez, Ibarra-Valdez, Rodriguez, & Dagdug, 2008; Graham & Field, 2007, 2008; Graham & Redies, 2010; Redies, 2007; Redies et al., 2007) with images of complex natural scenes (Burton & Moorhead, 1987; Field, 1987; Geisler, 2008; Ruderman & Bialek, 1994). This finding led to the hypothesis that many artists apply natural scene statistics when they create artworks (Graham & Redies, 2010; Redies, 2007). Another computational method for analyzing artworks is based on the analysis of histograms of oriented luminance gradients (HOGs; Bosch, Zisserman, & Munoz, 2008). This method allows calculating image properties such as self-similarity, complexity, and anisotropy (Amirshahi et al., 2012; Redies, Amirshahi, Koch, & Denzler, 2012). Results indicate that images of artworks exhibited in museums and images of natural scenes are highly self-similar, that is, subparts of the images have HOGs similar to the entire image (Amirshahi et al., 2012; Amirshahi, Redies, & Denzler, 2013). In other words, artworks possess a relatively high degree of self-similarity in comparison to other image categories (Braun, Amirshahi, Denzler, & Redies, 2014). In addition, large subsets of visual artworks have complexity values in an intermediate range (Braun et al., 2014). This finding is in line with the proposition by Berlyne (1974) that an intermediate level of complexity is associated with higher aesthetic appeal than low or high complexity on average, as experimentally confirmed by several studies (Forsythe, Nadal, Sheehy, Cela-Conde, & Sawey, 2011; Nadal, 2007). Furthermore, colored artworks are, in general, highly isotropic, that is, they contain luminance gradients of similar strength across all orientations (Braun et al., 2014; Koch, Denzler, & Redies, 2010; Melmer, Amirshahi, Koch, Denzler, & Redies, 2013). In our study, we measured the following SIPs: PHOG (Pyramid of HOGs) Self-Similarity, HOG Complexity, HOG Anisotropy, Aspect Ratio, Rule of Thirds, and various color measures. For an exact definition of these measures, see the Methods section. To measure these properties in images of artworks, we used the JenAesthetics database, which was introduced by Amirshahi, Denzler, et al. (2013). The data set consists of over 1,600 high-quality images of oil paintings of Western provenance. We used this type of artworks because it has been very popular over several centuries and thereby offers the opportunity to compare different art periods. For the rating experiment, we excluded more recent oil paintings (i.e., paintings with a year of origin later than 1935) and thereby eliminated paintings that are not intended to be visually pleasing. Furthermore, a previous study showed that representational paintings can be associated with more positive judgments on the dimensions of form, complexity, and regularity, as compared with abstract paintings (Marković, 2011).

A major concern regarding the analysis of the SIPs in this database was the age of some of the paintings, which can result in conservation artifacts, for example, a brownish film of varnish that may partially obscure color and luminance detail in the paintings (Bonaduce et al., 2012). We analyzed paintings in their present condition, in which museums made them available to the Google Art Project.

The subjective rating scores of the paintings have been obtained and analyzed in a previous study (Amirshahi, Hayn-Leichsenring, Denzler, & Redies, 2014a). Each image in this database was subjectively rated according to its *aesthetics* and its *beauty*. *Aesthetics* reflected the (“more objective”) artistic value of the respective image while *beauty* stands for the “subjective” liking by the individual participant. Given that there is some uncertainty with regard to the terminology (Augustin, Carbon, & Wagemans, 2012; Augustin, Wagemans, & Carbon, 2012), the terms were explicitly defined for the participants of the experiment (see section “Gaining of Subjective Rating Scores”). *Hedonic value* is used in the manuscript as a superordinate term for *aesthetics* and *beauty*. The present work extends the study by Amirshahi et al. (2014a) (a) by performing a deeper analysis of the subjective rating scores, (b) by analyzing the SIPs of the paintings, and (c) by investigating the relation between SIPs and the rating scores of the paintings.

Besides the search for universal aesthetic features, another interesting research topic is the identification of statistical properties that are characteristic for particular art periods. Marchenko, Tat-Seng, and Irina (2005) used computer algorithms based on the measurement of color temperature (warm or cold), color palette (primary or complimentary), and color contrasts (light or dark) to distinguish modern art from medieval art. Therefore, low-level statistics can provide informative cues about art periods. In the so-called *ontology-based disambiguation method*, Leslie, Chua, and Ramesh (2007) combined analyses of color measures and brush strokes with semantic high-level concepts to distinguish art periods; they achieved a higher performance than with the approach by Marchenko et al. The influence of higher level in categorization strategies has also been demonstrated by Wallraven et al. (2009). Hence, we did not only focus on the investigation of possible universal features of art images but expanded our effort toward an investigation of the usefulness of SIPs for allowing to differentiate art periods and subject matters.

In previous studies, rating scores on art paintings have been linked to interindividual differences between participants, such as personality traits (Furnham & Walker, 2001; Lyssenko, Redies, & Hayn-Leichsenring, 2016), expertise (Aluja, Garcia, & Garcia, 2004; Leder, Ring, & Dressler, 2013), demographic variables (Furnham & Walker, 2001) and other personal characteristics. For example, Mallon, Redies and Hayn-Leichsenring (2014) found that preferences differed between subgroups of participants depending on the SIPs of abstract paintings. Although the clustering of the participants was performed exclusively based on subjective evaluations, 46% of the clustering’s outcome was predicted by SIPs of the evaluated paintings. Güclütürk, Jacobs, and van Lier (2016) described that two clusters of participants differed in their liking of complexity in digital images. One group of participants showed increasingly lower liking rates for increasingly more complex images while another group showed the opposite pattern of preference. Bies, Blanc-Goldhammer, Boydston, Taylor, and Sereno (2016) found different preferences for clusters of participants who rated fractal patterns. These preferences correlated with specific patterns of fractal dimension, symmetry, and recursion in the stimuli. Together, these results indicate that different groups of people have different preferences for specific SIPs. Here, we investigated whether clusters of participants preferred certain SIPs over others in the images of the JenAesthetics database.

In summary, we provide a detailed statistical analysis of the subjective evaluations of the JenAesthetics database. Specifically, we connected the subjective rating scores with the SIPs. In addition, we reanalyzed the data set for individual art periods and subject matters to find out whether specific subsets of the JenAesthetics database differ in their SIPs or in the correlation between rating scores and the SIPs.

## Methods

### Data Set

**Figure 1.**
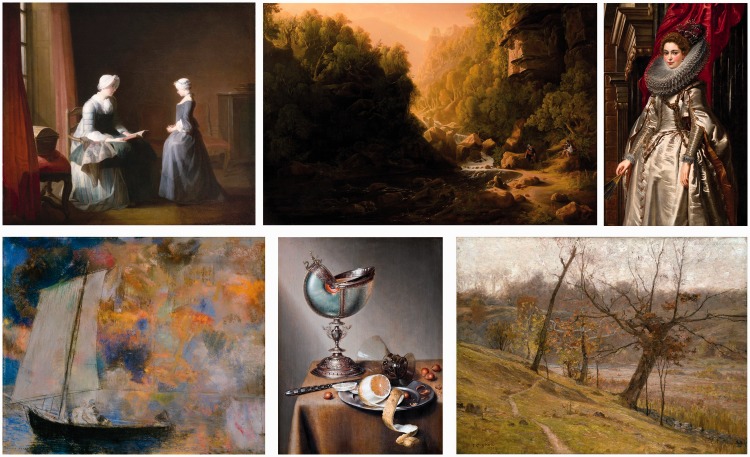
Example images from the JenAesthetics data set used in the subjective rating experiments. Upper row (from left to right): Jean-Baptiste-Siméon Chardin (1753), title: The Good Education, art period: Rococo, category: “scenes with persons”; Francis Danby (1830), The Mountain Torrent, Romanticism, “landscapes”; Peter Paul Rubens (1606), Portrait of Marchesa Brigida Spinola-Doria, Baroque, “portrait (one person)”; Lower row: Odilon Redon (1903), Flower Clouds, Symbolism, “seascape, port or coast”; Maerten Boelema De Stomme (1641), Still-Life with Nautilus Cup, Baroque, “still life”; Theodore Clement Steele (1893), The Bloom of the Grape, Impressionism, “landscapes.” All images are in the public domain.

We used the JenAesthetics data set (Amirshahi et al., 2012). This data set consists of 1,628 images of colored oil-paintings of Western provenance painted by over 400 artists. For technical reasons, we used 1,614 of the images only. The JenAesthetics data set is a subset of the Google Art Project database and therefore royalty free. All images were of high resolution (image size generally more than 3 MB; e.g., see Figure 1 images).

### Categorization of Paintings

As described in a technical report (Amirshahi et al., 2012), each image of the JenAesthetics database was categorized according to the art period and its subject matter. The categorization of the art periods followed standard textbooks on art and information available on the Wikipedia website. The data set contains works from 11 major art periods (Renaissance, Mannerism, Baroque, Rococo, Classicism, Romanticism, Realism, Impressionism, Symbolism, Post-Impressionism, and Expressionism). The categorization of subject matter of each painting is based on a subjective classification by two independent observers. Subject matters are *abstract*, *nearly abstract*, *landscapes*, *scenes with person(s)*, *still life*, *flowers or vegetation*, *animals*, *seascape, port or coast*, *sky*, *portrait (one person)*, *portrait (many persons)*, *nudes*, *urban scenes*, *buildings*, *interior scenes*, and *other subject matters*. Up to three subject matters were assigned to one particular painting. However, in our analysis, we used only the first (predominant) subject matter of each painting.

### Gaining of Subjective Rating Scores

In the present study, we used the rating scores for the JenAesthetics database, which were obtained in a previous study (Amirshahi et al., 2014a). In this study, participants rated the paintings on *aesthetic value*, *beauty*, *liking of color*, *liking of content*, *liking of composition*, *familiarity with the artist*, and *familiarity with the painting.* Before the experiment, participants were instructed that ratings on *aesthetics* should reflect the (“more objective”) artistic value of the respective image while *beauty* rating scores should reflect the “subjective” liking. In brief, the 1,614 paintings were rated in blocks of 163 randomly chosen images. Every painting was rated by 19 to 20 participants (131 participants in total). The rating experiment was performed with images that were reduced to a size of 800 pixels on the longest side (maximum size of 205 mm on the screen). Images were presented on a black screen (BenQ T221W widescreen monitor) that had been color calibrated using a colorimeter (X-Rite EODIS3 i1Display Pro). For a more detailed description of the experimental procedure, see Amirshahi et al. (2014a).

### Statistical Image Properties

For every painting, we calculated the following statistical properties using MATLAB 2008A:

1. PHOG Self-Similarity. Self-similarity implies that an object as a whole has a structure similar to its parts. In the present study, we calculated Self-Similarity using the PHOG method, as originally introduced by Bosch et al. (2008). In brief, the method follows a pyramid approach (Amirshahi et al., 2012): In a first step, the HOG feature (Dalal et al., 2005) for the entire image is calculated at the ground level (Level 0). The HOG feature represents the mean strength of the luminance gradients binned in 16 equally sized orientations that cover all orientations in the image. Second, the image was divided into four rectangles of the same size (Level 1), and the HOG features were calculated for each subimage. Then, each of the four rectangles was again divided into equal rectangles (Level 2), and the HOG features were calculated for the resulting 16 subimages as well. We took this approach up to Level 3. Then, we compared the HOG features of the entire image on Level 0 with the HOG features of the rectangles on the third level using the Histogram Intersection Kernel (Barla et al., 2002). A detailed description of the method (Amirshahi et al., 2012) can be found in the Appendix to Braun et al. (2014). This measure ranges from 0 (*no self-similarity*) to 1 (*maximal self-similarity*).
2. HOG Complexity. Several recent studies confirmed the importance of complexity in aesthetic perception (Bies et al., 2016; Forsythe et al., 2011; Jacobsen & Hofel, 2002; Rigau, Feixas, & Sbert, 2008). Here, we defined Complexity as the total strength of all oriented gradients in a painting (Redies et al., 2012).
3. HOG Anisotropy. Anisotropy is a measure for the heterogeneity (variance) of luminance gradients across orientations in a particular image. High values indicate that some orientations of gradients are represented more strongly than others in the HOGs, while low values imply that the luminance gradients are uniformly distributed across all orientations (Redies et al., 2012). Schweinhart and Essock (2013) showed that art paintings of different subject matters (e.g., landscapes and faces) tend to be less anisotropic even though, in real-world photographs, the pattern of anisotropy differs considerably between landscapes and faces. Possibly, artists over regularize the structure in their paintings by imposing the natural-scene horizontal effect in other types of subject matter.
4. Aspect Ratio. Although there is no evidence for an overall hedonic preference of a certain format of paintings (McManus, 1980; Russell, 2000), the aspect ratio has been linked previously to the aesthetic preference of paintings depicting specific subject matters, for example, landscapes or portraits (Palmer, Schloss, & Sammartino, 2013). Therefore, we included this measure because it might be related to preferences for images from specific art periods or of specific subject matters.
5. Rule of Thirds. We measured the degree, to which images structure followed the Rule of Thirds (see Amirshahi, Hayn-Leichsenring, Denzler, & Redies, 2014b). The Rule of Thirds implies that the focus point of a painting should be placed along one of the third lines to yield aesthetically pleasing results. In the present study, the focus point was determined based on the graph-based visual saliency method, as described by Mai, Le, Niu, and Liu (2011).
6. Color measures. In addition, we calculated the three color measures of the HSV color space (color hue, color saturation, and color value), which have been regularly used in aesthetic quality assessment (e.g., see Datta et al., 2006; Palmer et al., 2013). All color measures range from 0 to 1. Color hue ranges from red (value = 0), yellow, green, cyan, blue, to magenta (value = 1). For color saturation, higher values stand for higher saturated images and for color hue, higher values stand for brighter images.

### Clustering of the Paintings and Participants

We divided the paintings with the k-means clustering method according to their mean subjective rating scores on *aesthetics* and *beauty*. Clustering allowed allocation of paintings into three subgroups that resembled each other according to correlation patterns. This number of clusters was judged to be close to optimal based on the elbow criterion, a computational feature in plots of the sum of squared errors. We used the same method (but based on correlations of subjective ratings with SIPs) for the clustering of the participants.

## Results

### Analysis of SIPs

#### Correlation between SIPs

The measured SIPs correlate with each other (see Supplementary Table 1 for a detailed analysis of correlations of SIPs within the JenAesthetics database). For example, there is a high correlation between Self-Similarity and Anisotropy (*r* = −483, *p* < .001). Bearing this in mind, we performed detailed statistical analyses on SIPs over paintings and over participants.

### Analysis Over Paintings

#### Correlation of SIPs with year of origin

First, we investigated whether the SIPs of the paintings changed over the years. Therefore, we correlated the year of origin of the paintings with the SIPs. We found significant correlations for most of the SIPs, namely for Self-Similarity (Pearson’s *r* = .204, *p* < .001), Complexity (*r* = .127, *p* < .001), Anisotropy (*r* = .090, *p* < .001), Aspect Ratio (*r* = −.182, *p* < .001), Rule of Thirds (r = −.107, *p* < .001), Color Saturation (*r* = −.287, *p* < .001), and Color Value (*r* = .425, *p* < .001). Therefore, image statistics changed over time. More recent oil paintings are more self-similar and more complex. Also, they possess a higher degree of Anisotropy and show different colors.

#### SIPs in paintings from different art periods

We then analyzed whether paintings from the various art periods differed in their SIPs.

The results were entered into a one-way analysis of variance (ANOVA) considering art period as between-subject factor. Results revealed a significant effect of all measured SIPs: Self-Similarity, *F*(10, 1603) = 11.344; *p* < .001, Complexity, *F*(10, 1603) = 17.685; *p* < .001, Anisotropy, *F*(10, 1603) = 4.596; *p* < .001, Aspect Ratio, *F*(10, 1603) = 11.742; *p* < .001, Rule of Thirds, *F*(10, 1603) = 7.040; *p* < .001, Color Hue, *F*(10, 1603) = 16.319; *p* < .001, Color Saturation, *F*(10, 1603) = 20.184; *p* < .001, and Color Value, *F*(10, 1603) = 51.913; *p* < .001. Therefore, all measured SIPs showed differences over the art periods analyzed (see Supplementary Table 2 for descriptive statistics and Supplementary Table 3 for results of a multivariable linear regression analysis that studied the interaction between the SIPs in one model and their affiliation to a specific art period and Supplementary Figure 1 for a plot of the LOESS curve fittings for *aesthetics* and *beauty*). In addition, we performed a multinomial multivariable regression analysis for art period with all SIPs. Results showed that all SIPs are significant predictors of the art period affiliation. For example, an increase of saturation of 0.1 increases the probability of belonging to the art period Renaissance by 79.8% as compared with the reference art period Impressionism (see Table 1 for complete results).

**Table 1.** Odds Ratios From a Multinomial Multivariable Regression Analysis for Art Period With SIPs With ‘Impressionism’ as the Reference for Art Period. Displayed Are the Odds Ratios Per Change of 0.1 in Values of the Respective SIP.

	Renaissance	Mannerism	Baroque	Rococo	Classicism	Romanticism	Realism	Symbolism	Post-Impressionism	Expressionism
Self-Similarity	0.232**	0.171**	0.456*	0.778	0.452	1.701	0.566	0.44	2.372*	1.475
Complexity	0.993*	1.012*	0.994*	0.988*	0.968**	0.989**	0.987**	0.999	0.999	1.003
Anisotropy	1E-13*	1E-13*	1E-13*	1.47E+185	1E-13	9.293E+157	1E-13	1E-13	4.284E+114	1.163E+257
Rule of Thirds	0.495*	0.333*	0.826	1.004	0.873	1.192	1.457	1.916	0.603	0.347
Color Hue	0.752	0.878	0.693**	0.633**	0.666*	0.633**	0.746*	0.838	1.27*	1.725**
Color Saturation	1.798**	1.472*	1.408**	1.337*	1.595**	1.188	1.328*	1.44*	1.414**	2.49**
Color Value	0.471**	0.296**	0.384**	0.523**	0.476**	0.484**	0.676**	0.991	0.956	1.082
Aspect Ratio	1.025	1.163*	0.948	0.923	0.951	0.816**	0.81**	1.095	0.981	1.063

* *p* < . 05. **p < .001.

#### SIPs in paintings with different subject matters over art periods

Next, we compared the SIPs of landscape and portrait paintings between different art periods. We used landscape and portrait paintings because these subject matters were the most numerous within the JenAesthetics database and, additionally, they were also common to all art periods.

The results were entered into a one-way ANOVA considering art period as between-subject factor. For portrait paintings, results revealed a significant effect of Complexity, *F*(10, 428) = 8.366; *p* < .001, Anisotropy, *F*(10, 428) = 2.481; *p* < .01, Aspect Ratio, *F*(10, 428) = 2.522; *p* < .01, Rule of Thirds, *F*(10, 428) = 4.051; *p* < .001, Color Hue, *F*(10, 428) = 3.272; *p* < .001, Color Saturation, *F*(10, 428) = 4.066; *p* < .001, and Color Value, *F*(10, 428) = 18.486; *p* < .001. Only Self-Similarity, *F*(10, 428) = 1.686, ns, had no effect. Therefore, only Self-Similarity did not differ significantly in portrait paintings over the art periods.

For landscape paintings, results revealed a significant effect of Self-Similarity, *F*(9, 168) = 3.411; *p* = .001, Complexity, *F*(9, 168) = 6.326; *p* < .001, Rule of Thirds, *F*(9, 168) = 3.054; *p* < .01, Color Hue, *F*(9, 168) = 7.089; *p* < .001, Color Saturation, *F*(9, 168) = 2.418; *p* < .05, and Color Value, *F*(9, 168) = 6.239; *p* < .001. In landscape paintings, Anisotropy, *F*(9, 168) = .844, ns, and Aspect Ratio, *F*(9, 168) = .980, ns, did not differ significantly between art periods.

In conclusion, all SIPs show changes throughout the art periods, except for Self-Similarity, which does not change in portrait paintings, and Anisotropy and Aspect Ratio, which are unaltered in landscape paintings (see Supplementary Table 4 for the descriptive statistics on landscape paintings and portrait paintings for every art period). To detect the influence of SIPs and subject matter (independent variables) including their interaction on *aesthetics* (dependent variable), a multivariable linear regression model was fitted (see Supplementary Table 5 for results and Supplementary Table 6 for descriptive statistics on all subject matters).

#### Subjective rating scores and year of origin of the paintings

The year of origin of the paintings correlated positively with *beauty* (*r* = .168, *p* < .001), liking of color (*r* = .054, *p* < .05), liking of content (*r* = .306, *p* < .001), liking of composition (*r* = .092, *p* < .001), familiarity with the artist (*r* = .280, *p* < .001), and familiarity with the painting (*r* = .050, *p* < .05). Interestingly, evaluation on *aesthetics* showed no correlation (*r* = −.011, *p* = .673). We performed a Fisher transform to investigate whether differences of correlations between *aesthetics* or year of origin and *beauty* or year of origin were significant. To this aim, we converted Pearson’s *r* to Fisher’s *z* and computed the confidence interval at 99%. Results showed a significant difference of the correlations. This is particularly striking because subjective evaluation on *beauty* and *aesthetics* (see Introduction for definition of the terms) are highly correlated (*r* = .772, *p* < .001). Therefore, participants personally preferred newer paintings while the ascribed (“objective”) artistic value remained stable over time.

#### General analysis of subjective rating scores in different art periods

**Figure 2.**
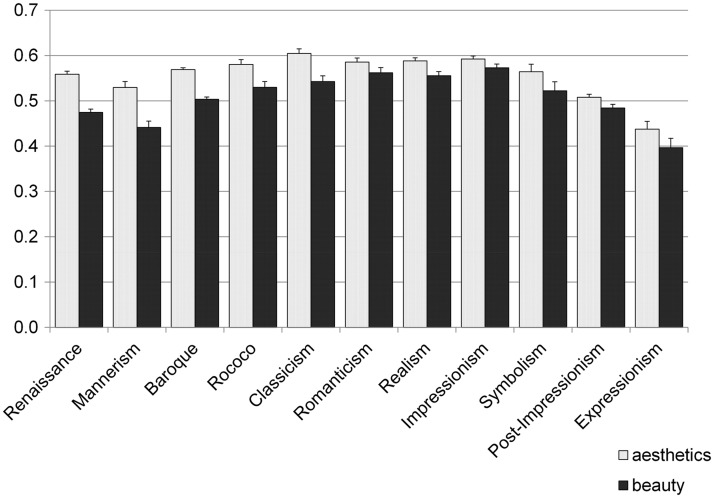
Mean scores for the subjective evaluation on *aesthetics* and *beauty* for images from different art periods. Whiskers indicate standard errors of the mean.

Next, we investigated general differences of subjective rating scores between the art periods. Although each art period is obviously related to the year of origin, participants might be less familiar with certain art periods than with others and, therefore, they might systematically prefer paintings from particular art periods, irrespective of their year of origin. The subjective rating scores were entered into a one-way ANOVA considering art period as between-subject factor. Results revealed a significant effect of scores on *aesthetics*, *F*(11, 1605) = 18.249; *p* < .001, as well as on scores on *beauty*, *F*(11, 1605) = 17.155; *p* < .001. Thus, although we found no general effect of the year of origin on *aesthetics*, there are still differences in appreciation for the different art periods (see Figure 2 for mean scores for the respective art periods).

#### Subjective rating scores and SIPs

We investigated the relation between subjective rating scores and the SIPs. To this aim, two stepwise (backward elimination) multiple linear regression analyses were run. The subjective rating scores (*aesthetics* or *beauty*) were the dependent variables while standardized (z-transformed) values for SIPs were considered independent variables. Results were controlled with the post hoc Holm–Bonferroni method. Four of the SIPs predicted subjective ratings on *aesthetics*, *F*(4, 1609) = 14.465; *p* < .001. These were Aspect Ratio (β = − .129, *t* = −5.044, *p* < .001), Self-Similarity (β = −.081, *t* = −3.014, *p* < .05), Color Value (β = .085, *t* = 3.207, *p* = .001), and Complexity (β = −067, *t* = −2.514, *p* < .05). The subjective ratings on *beauty* can be predicted in a similar way, *F*(3, 1610) = 40.577; *p* < .001, by Aspect Ratio (β = −.199, *t* = −8.048, *p* < .001), Color Value (β = .188, *t* = 7.380, *p* < .001), and Self-Similarity (β = −.093, *t* = −3.765, *p* < .001). In addition, in a correlation analysis after Fisher’s *z* transformation, we found that correlations with Aspect Ratio and Color Value only were significantly different for the hedonic ratings. Therefore, *aesthetics* and *beauty* ratings showed an overall similar pattern of correlations with the SIPs.

Subjective rating scores on preferences for *color*, *composition*, and *content* correlated with several SIPs (see Table 2 for details).

**Table 2.** Correlation Coefficients (Pearson’s *r*) for Subjective Rating Scores and Statistical Image Properties.

Subjective scale	Self-Similarity	Complexity	Anisotropy	Aspect Ratio	Rule of Thirds	Color Hue	Color Saturation	Color Value
*aesthetics*	−.066**	−.070**	.065**	−.137**	−0.04	−0.013	−0.028	.081**
*beauty*	−0.018	**−**0.015	.110**	−.237**	−.091**	**.051** *	−.111**	.214**
*color*	−.067**	0.012	.100**	−.182**	−.125**	.069**	0.044	.238**
*composition*	**−**0.041	**−**0.037	.068**	−.219**	**−.052** *	**−**0.016	−0.043	.108**
*content*	**.055** *	**.051** *	.124**	−.286**	−.125**	.076**	−.195**	.306**

* *p* < . 05. **p < .001. Bold values represent *p* < .05 and are additionally marked with one or two asterisks, respectively.

#### Subjective rating scores and SIPs in paintings from different art periods

Next, we investigated correlations between subjective rating scores and the SIPs for different art periods. Here, we focused on the subjective rating scores for *aesthetics* and *beauty* and investigated only art periods with more than 30 paintings in the database (see Table 3). Interestingly, the number of correlations of subjective rating scores differed between art periods. There were no correlations with the SIPs for Mannerism, Classicism, Romanticism, Symbolism, and Expressionism, and few correlations for Renaissance, Realism, Impressionism, Post-Impressionism, and Expressionism. In contrast, Baroque and Rococo showed correlations of subjective rating scores with several SIPs.

**Table 3.** Correlation Coefficients (Pearson’s *r*) for Subjective Rating Scores and SIPs for Different Art Periods.

Art period	Subjective Scale	Self-Similarity	Complexity	Anisotropy	Aspect Ratio	Rule of Thirds	Color Hue	Color Saturation	Color Value
Renaissance *n* = 110	*aesthetics*	−.128	−.192*	.106	−.082	−.066	.153	.078	.177
	*beauty*	−.009	−.099	.005	−.218*	−.092	.082	.103	.181
Mannerism *n* = 36	*aesthetics*	−.062	.075	−.124	−.121	−.094	.069	−.041	.113
	*beauty*	−.250	−.038	−.103	−.090	−.048	.066	.025	−.078
Baroque *n* = 503	*aesthetics*	−.011	−.048	.079	−.255**	−.113*	**.102** *	−.087	.250**
	*beauty*	−.032	−.043	.131**	−.325**	−.144**	**.100** *	−.136**	.326**
Rococo *n* = 93	*aesthetics*	−.179	.079	.139	−.378**	−.431**	**.223** *	−.290**	.547**
	*beauty*	−.134	.071	.182	−.437**	−.411**	**.259** *	−.416**	.578**
Classicism *n* = 70	*aesthetics*	.150	−.014	−.172	−.014	.015	.085	.010	.022
	*beauty*	.157	.089	−.179	−.050	−.058	.038	−.024	.115
Romanticism *n* = 142	*aesthetics*	−.156	−.051	.062	−.024	−.068	.033	.066	.022
	*beauty*	−.120	−.083	.136	−.163	−.119	.027	−.037	.098
Realism *n* = 178	*aesthetics*	−.144	−.047	.112	.053	.006	.074	.020	.120
	*beauty*	−.202**	.019	.141	−.039	.000	.133	.047	.168*
Impressionism *n* = 206	*aesthetics*	.028	−.062	.099	−.100	−.038	.006	.044	**.160** *
	*beauty*	.073	−.053	.107	−.140*	−.071	.093	−.014	.230**
Symbolism *n* = 42	*aesthetics*	.007	−.005	−.006	−.057	.038	−.149	.068	−.284
	*beauty*	.037	−.003	.119	−.131	−.051	−.142	.143	−.182
Post-Impress. *n* = 202	*aesthetics*	.030	.211**	.045	−.082	−.097	−.088	−.026	.082
	*beauty*	.038	.236**	.082	−.152*	−.102	−.040	−.031	.120
Expressionism *n* = 32	*aesthetics*	−.119	−.147	.201	.220	.074	−.004	−.233	−.310
	*beauty*	−.169	−.024	.235	.098	.054	.031	−.105	−.291

* *p* < . 05. **p < .001. Bold values represent *p* < .05 and are additionally marked with one or two asterisks, respectively.

These results provide evidence that the evaluation of artworks from certain art periods (Baroque, Rococo) by our observers is correlated with particular SIP patterns, while this is not the case for artworks from other art periods (Mannerism, Classicism, Romanticism, Symbolism, and Expressionism).

#### Subjective rating scores and SIPs in paintings with different subject matters

We investigated correlations between subjective rating scores and the SIPs for different subject matters that comprised more than 30 paintings. Again, we focused on subjective rating scores for *aesthetics* and *beauty*. In paintings of *landscapes*, *flowers or vegetation*, *seascapes*, and *portraits with many persons*, there were no correlations of subjective rating scores with the SIPs, while paintings with other subject matters showed at least some correlations. Particularly, the hedonic evaluation of paintings of buildings seems to be connected with SIPs (especially with Self-Similarity, Color Saturation, and Color Value; see Table 4 for detailed results). We conclude that hedonic ratings correlate with SIPs for artworks of certain subject matters only.

**Table 4.** Correlation Coefficients (Pearson’s *r*) for Subjective Rating Scores and SIPs for Different Subject Matters.

Art period	Subjective Scale	Self-Similarity	Complexity	Anisotropy	Rule of Thirds	Color Hue	Color Saturation	Color Value	Aspect Ratio
Landscapes *n* = 179	*aesthetics*	−.166*	−.031	−.025	−.059	−.043	.121	.059	−.045
	*beauty*	−.154*	.030	−.040	−.086	.024	.116	.036	−.060
Scenes with persons *n* = 490	*aesthetics*	−.070	−.085	.002	−.011	−.020	−.071	−.036	.015
	*beauty*	−.088	−.068	.053	.013	.000	−.098*	.017	−.052
Still life *n* = 66	*aesthetics*	−.031	.034	−.071	−.148	−.222	**.249** *	−.172	.144
	*beauty*	−.052	.059	−.023	−.182	−.130	**.257** *	−.085	.202
Flowers or vegetation *n* = 43	*aesthetics*	−.224	−.030	−.262	.055	.017	.135	−.040	.126
	*beauty*	−.365*	−.112	−.146	−.026	.241	.017	.100	.048
Seascape, port, or coast *n* = 96	*aesthetics*	−.125	−.125	−.050	.022	−.107	.027	−.012	−.105
	*beauty*	−.211*	−.164	.013	.014	−.032	.103	−.051	−.076
Portrait (one person) *n* = 439	*aesthetics*	−.097*	−.109*	.038	.045	.002	.062	−.022	−.079
	*beauty*	−.066	−.095*	.063	−.004	.047	−.008	**.107** *	−.150**
Portrait (many persons) *n* = 79	*aesthetics*	−.060	−.083	.209	.037	.057	.036	−.020	.090
	*beauty*	.017	.013	**.228** *	.003	−.001	.145	.121	.069
Urban scene *n* = 65	*aesthetics*	−.315*	−.186	**.311** *	.151	.153	−.190	.119	−.079
	*beauty*	−.172	−.145	.209	.163	.206	−.178	.141	−.045
Building *n* = 36	*aesthetics*	−.427**	−.217	.146	.157	−.421*	**.417** *	−.435**	−.271
	*beauty*	−.503**	−.053	.094	.257	−.272	**.361** *	−.446**	−.313

* *p *< . 05. **p < .001. Bold values represent *p* < .05 and are additionally marked with one or two asterisks, respectively.

#### Clustering based on subjective rating scores on aesthetics and beauty

To gain insight into the evaluations on artistic value (*aesthetics*) and subjective liking (*beauty*) and their relation to SIPs, we clustered the paintings (for a description of the method, see Methods section). We calculated the standard deviation of the mean values of each image from the mean of each cluster for two to six clusters. An elbow criterion provided evidence that three is the optimal number of clusters. The clusters consisted of 750, 481, and 383 paintings, respectively. While Cluster 1 (*aesthetics*: *M* = .56, *beauty*: *M* = .50) and Cluster 3 (*aesthetics*: *M* = .44, *beauty*: *M* = .37) had significantly higher ratings on *aesthetics* than on beauty, Cluster 2 (*aesthetics*: *M* = .67, *beauty*: *M* = .66) consisted of paintings that had a similar mean value of ratings on *aesthetics* and *beauty*. Next, we asked whether the three clusters had different mean values of SIPs (see Table 5 for results). We found significant differences of mean values between Clusters 1 and 2 for Anisotropy (two-samples *t* test: *T*(1229) = −2.966, *p* < .01), Rule of Thirds, *T*(1229) = 2.900; *p* < .01, Color Saturation, *T*(1229) = 4.308; *p* < .001, Color Value, *T*(1229) = −6.838; *p* < .001, and Aspect Ratio, *T*(1229) = 4.210; *p* < .001, as well as between Clusters 2 and 3 for Anisotropy, *T*(862) = 3.474; *p* < .001, Rule of Thirds, *T*(862) = −3.155; *p* < .01, Color Saturation, *T*(862) = −3.481; *p* < .001, Color Value, *T*(862) = 6.153; *p* < .001, and Aspect Ratio, *T*(862) = −7.216; *p* < .001. Between Clusters 1 and 3, mean values differed for Aspect Ratio, *T*(1131) = −3.879; *p* < .001, only. Therefore, it can be concluded that paintings in Cluster 2 (for which subjective rating scores of *aesthetics* and *beauty* had a similar mean value) differ in their mean value for several SIPs.

**Table 5.** Mean Values of the SIPs for Clusters 1 to 3.

	Self-Similarity	Complexity	Anisotropy	Rule of Thirds	Color Hue	Color Saturation	Color Value	Aspect Ratio
Cluster 1	0.868	7.813	0.00014906	0.216	0.221	0.390	0.392	1.023
Cluster 2	0.867	7.742	0.00015941	0.207	0.234	0.355	0.448	0.946
Cluster 3	0.872	8.172	0.00014594	0.219	0.224	0.388	0.389	1.103

In addition, we performed a stepwise (backward elimination) multivariable linear regression analysis over all paintings (from all clusters). The mathematical difference *aesthetics* minus *beauty* was considered as dependent variable while standardized (z-transformed) values for SIPs were considered independent variables. Results were controlled with the post hoc Holm–Bonferroni method. Four of the SIPs predicted the difference between subjective ratings, *F*(4, 1609) = 67.410; *p* < .001. These were Color Value (β = −.238, *t* = −9.812, *p* < .001), Aspect Ratio (β = .191, *t* = 7.958, *p* < .001), Color Hue (β = −.092, *t* = −3.931, *p* < .001), and Anisotropy (β = −.072, *t* = 3.080, *p* = .01). We conclude that differences in evaluation on *aesthetics* and *beauty—*or the ascribed artistic value and the subjective liking—might depend on SIPs.

**Figure 3.**
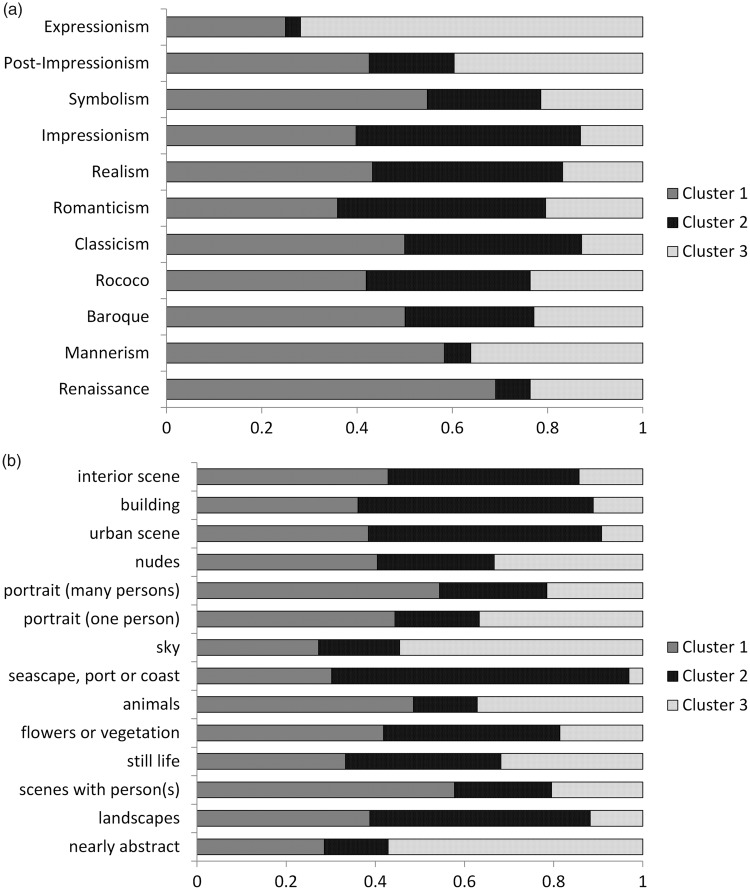
Analysis of image clustering. Displayed are the percentage number of paintings assigned to the three clusters for (a) art periods and (b) subject matters. Clustering of the images was based on their mean subjective rating scores on *aesthetics* and *beauty*. Cluster 1 consisted of 750 images (46.5% of all images), Cluster 2 consisted of 481 images (29.8%), and Cluster 3 consisted of 383 images (23.7%).

After clustering, 29.8% of the paintings were assigned to Cluster 2. However, when analyzed over art periods, we found that the percentage of paintings assigned to Cluster 2 differed. For Renaissance (C2 = 7.2%), Mannerism (C2 = 5.5%), and Expressionism (C2 = 3.1%), there were only few paintings in Cluster 2, while for Romanticism (C2 = 43.7%) and for Impressionism (C2 = 47.1%), Cluster 2 comprised numerous paintings. Therefore, the artistic value and subjective liking that participants ascribed to romantic and impressionist paintings were similar. Participants ascribed artistic value to paintings from Renaissance, Mannerism, and Expressionism although they did not like these paintings subjectively to the same degree (see Figure 3(a) for detailed results).

Analyzed over subject matters, Cluster 2 contained only a few paintings from the categories *nearly abstract* (C2 = 14.2%), *animals* (C2 = 14.2%), *sky* (C2 = 18.2%), and *portraits with one person* (C2 = 18.9%), while paintings in the categories of *landscapes* (C2 = 49.4%), *seascapes* (C2 = 66.7%), *urban scenes* (C2 = 52.3%), and *buildings* (C2 = 52.7%) were more numerous. In summary, especially in paintings of wide scenes, the subjective rating scores on *aesthetics* and *beauty* were similar (see Figure 3(b) for detailed results).

### Analysis Over Participants

#### General analysis

For the analysis over participants, we focused solely on the subjective rating scores on *beauty* (reflecting the individual liking of the images), because we were interested in the participants’ personal taste and not in what they considered to be generally *aesthetic*. Overall, the mean of the subjective rating scores on *beauty* was *M* = .416 (*SD* = .338). Not surprisingly, participants that reported interest in arts gave higher ratings than participants that did not report interest in arts (interested: *M* = .431, *SD* = .081; noninterested: *M* = .386, *SD* = .015; two-samples *t* test: *T*(116) = 2.447, *p* < .05).

#### Clustering over SIPs

We calculated Pearson’s correlation coefficient for subjective rating scores on *beauty* with SIPs of the rated paintings for every single participant (see Supplementary Table 7 for a complete analysis).

As the correlations were heterogeneous among the participants, that is, groups of participants showed a particular pattern of correlations with certain SIPs, we divided the participants with the k-means clustering method according to their respective correlation pattern. For the clustering, we calculated the standard deviation of the mean values of each participant from the mean of each cluster for two to seven clusters. Then, we calculated the sum of squares for two to seven clusters (SS_2_ = .066, SS_3_ = .054, SS_4_ = .052, SS_5_ = .046, SS_6_ = .042, SS_7_ = .039). The elbow criterion provided evidence that a number of three clusters is optimal. These three clusters consisted of 29, 37, and 65 participants, respectively.

**Figure 4.**
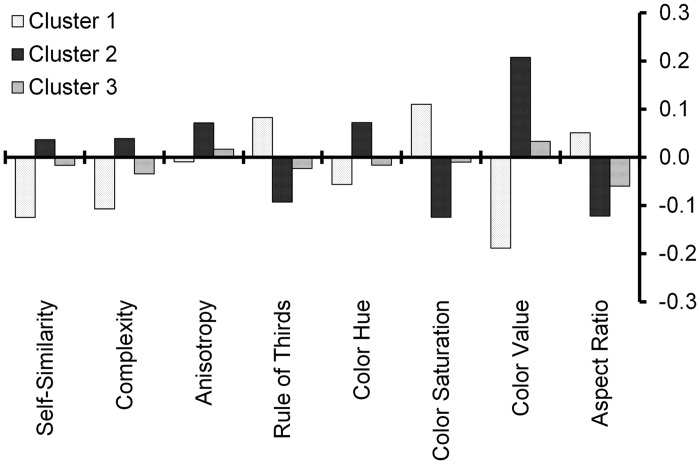
Analysis of participant clustering. Mean correlations (Pearson’s *r*) of subjective rating scores on *beauty* with SIPs for each cluster. Clustering of participants was based on the correlation of *beauty* ratings with SIPs.

To further justify our clustering, we calculated the mean correlations of subjective rating scores on *beauty* with SIPs for each cluster. Participants in two of three clusters (Clusters 1 and 2) showed a strong relation between subjective rating scores and SIPs, while SIPs had only a small effect on subjective rating scores of participants in Cluster 3 (see Figure 4 for results).

The results were entered into a one-way ANOVA considering cluster affiliation as between-subject factor. Results revealed a significant effect of Self-Similarity, *F*(2, 128) = 40.061; *p* < .001, Complexity, *F*(2, 128) = 32.340; *p* < .001, Anisotropy, *F*(2, 128) = 10.269; *p* < .001, Aspect Ratio, *F*(2, 128) = 27.794; *p* < .001, Rule of Thirds, *F*(2, 128) = 53.073; *p* < .001, Color Hue, *F*(2, 128) = 26.141; *p* < .001, Color Saturation, *F*(2, 128) = 71.873; *p* < .001, and Color Value, *F*(2, 128) = 194.277; *p* < .001. Post hoc comparisons using the Tukey HSD test indicated a significant difference between individual clusters for mean values of each SIP analyzed.

## Discussion

### Statistical Image Properties

One of the central questions in experimental aesthetics is whether there is a “universal beauty” in artworks, natural scenes, and faces. In recent years, this question has been studied by novel computational methods that allow measuring specific image properties. In this context, the comparison between aesthetic and ordinary (nonaesthetic) images is of particular interest. For example, Braun et al. (2014) investigated SIPs in different categories of images. They showed that artworks, as a category of images that are created to be aesthetic, exhibit a relatively high degree of Self-Similarity, a low degree of Anisotropy, and an intermediate degree of Complexity. In these statistical measures, art paintings differ from other image categories like photographs of natural scenes, urban scenes, faces, and simple objects, as well as advertisements, on average. Here, we investigated the SIPs of oil paintings. We asked whether SIPs differed between art periods and between depicted subject matters in this set of paintings. Therefore, we investigated these subgroups of paintings separately. Our results show that, for each SIP, there are significant differences between art periods (see SIPs in paintings from different art periods section). Therefore, we did not obtain evidence that some SIPs are stable over all art periods investigated. However, in a more detailed analysis, we found that Anisotropy did not differ significantly over landscape paintings while it differs between art periods in portrait paintings. Conceivably, Anisotropy is uniformly high in landscape paintings because horizontal orientations (e.g., horizon) and vertical orientations (e.g., trees) predominate in real-natural scenes. The opposite pattern was observed for PHOG Self-Similarity, which differed in landscape paintings over art periods but did not differ over portrait paintings (see SIPs in paintings with different subject matters over art periods section). Our results are similar to findings by Redies et al. (2007) who showed that the Fourier slopes of grayscale art portraits did not resemble those of face photographs but of natural scenes. They concluded that artists portray faces not by mere copying of their real-world counterparts but by using specific divergent image statistics. In the present study, we provide another example for the usage of particular image properties (i.e., PHOG Self-Similarity) in artistic renderings of faces.

Fourier slope and Self-Similarity are correlated in artworks (Braun et al., 2014). Not only do artists portray faces with statistics divergent from real-world faces, but they also use relatively stable statistics to do so. Overall, our results provide evidence that artists from all art periods endow oil paintings of particular subject matters with similar image properties.

### Subjective Rating Scores

The terminology that relates to aesthetic experience has been widely discussed in aesthetics research (Augustin, Carbon, et al., 2012; Augustin, Wagemans, et al., 2012). Here, we distinguish between *aesthetics* and *beauty* ratings. Participants were instructed that ratings on *aesthetics* should reflect the (“more objective”) artistic value of the respective image while *beauty* rating scores should reflect the subjective liking (see Introduction and Gaining of Subjective Rating Scores sections). Rating scores differed between art periods, with classicist paintings evaluated as most *aesthetic* and impressionist paintings evaluated as most *beautiful* (see Figure 2). Overall, participants subjectively preferred more recent paintings while their rating of ascribed artistic value (i.e., *aesthetics*) was relatively stable over the centuries (see Subjective rating scores and year of origins of the paintings section). Consequently, we propose that, on the one hand, contemporary observers prefer more recent oil paintings, possibly because they are more familiar with them. On the other hand, the observers appreciated the artistic expertise of painters in the different periods to the same degree. This is in line with the notion that the skills of artists are more or less stable on average over the centuries. Unlike artistic skills, the taste of individual observers changes over time and, therefore, contemporary participants prefer more modern paintings in general. This preference might be based on a mere exposure effect for more recent paintings or, alternatively, on a shared preference for similar semantic content. Previously, it had been shown that visual preferences can be based on the semantic content of stimuli and shared semantic interpretations can lead to shared preferences (Vessel & Rubin, 2010).

Interestingly, ratings on *aesthetics* and *beauty* were quite similar for impressionist paintings. Therefore, impressionist paintings are valued artistically (*objectively*) to the same degree as *subjectively*. Again, this result may be explained by the greater familiarity of the observers with impressionist paintings in comparison to paintings from other art periods.

Focusing on the subject matter, ratings on *aesthetics* and *beauty* were similar in paintings of large-vista scenes (like landscapes, seascapes, urban scenes, and buildings). Interestingly, for some subject matters, *aesthetics* scores were higher than subjective liking (*beauty*) scores, especially for portrait and animal paintings. This difference might be explained by the content of the images. For example, in portrait paintings, participants might appreciate their artistic value, but they do not like the image subjectively, possibly because the liking or disliking of the displayed person might have an effect on this rating. Last but not least, no subset of images was rated as highly *beautiful* but not as *aesthetic.*

### Subjective Rating Scores and SIPs

It has been shown that specific SIPs are related to the hedonic value of abstract art paintings (Mallon et al., 2014). In the present, more detailed study, we show that rating scores on artistic value correspond to a slightly greater extent with SIPs than rating scores on subjective liking (see Table 1). It is not surprising that the more objective ratings on *aesthetics* correlate stronger with *objective* image properties, such as specific SIPs.

In addition, we clustered paintings according to their ratings on *aesthetics* and *beauty*. We found that differences in rating scores correlated with specific SIPs, especially with Anisotropy, Rule of Thirds, Color Saturation, and Color Value (see Clustering based on subjective rating scores on *aesthetics* and *beauty* section). This result points to an interaction between objective properties (SIPs) and the subjective evaluation of the images. However, these differences might also be explained by other factors (e.g., preference for specific contents or styles that coincide with certain SIPs in the paintings). In addition, this finding does not hold for all art periods because hedonic evaluation is not correlated with SIPs for Mannerism, Romanticism, and Symbolism. Hence, the subjective liking of paintings from these periods must be driven by other factors.

We observed similar differences for subject matters (see Table 3). Here, we found that ratings of each subject matter correlated—at least weakly—with specific SIPs. Especially the rating of *buildings* showed relatively high correlations with Self-Similarity, Color Saturation, and Color Value.

### Analysis Over Participants

In addition to our analysis of paintings, we also searched for similarities in the evaluations by the participants. In previous research, rating scores on art have been linked to expertise (Leder et al., 2013), personality traits (Lyssenko et al., 2016), and other characteristics of participants. Furthermore, it has been demonstrated that individuals exhibit stable patterns of preference for fractal-like characteristics across different image types (Spehar, Walker, & Taylor, 2016). In the present study, we focused on preferences for SIPs in groups of participants. We analyzed three clusters of participants. Affiliation to a certain cluster reflects a specific rating pattern that correlates, in turn, with preferences for images with specific SIPs. Two of the clusters showed multiple correlations of rating scores on *beauty* with particular SIPs (Figure 4). In Clusters 1 and 2, Self-Similarity, Complexity, Anisotropy, Color Saturation, and Color Value of the painting had an effect on the subjective preference, while in Cluster 3, the SIPs were not correlated with preferences (see Clustering over SIPs section). Hence, about two thirds of the participants were (perhaps unconsciously) sensitive to image statistics. A possible reason for this finding is that paintings of similar content or art style have similar image statistics. Therefore, a coherent taste may coincide with a preference for similar image statistics. Notably, the third group of participants showed only very few correlations of subjective ratings with SIPs. Perhaps, these participants possessed a rather incoherent taste or, possibly, a taste for different image features or statistical properties that have not been measured in the present study. Alternatively, their preference for paintings might be driven more by cognitive than by sensory factors, that is, these participants possibly focus more on image content than on artistic composition. It will be of interest to study the differences between such groups of participants in future research in more detail.

### Limitations

In the presented study, we used images of oil paintings as stimuli and, therefore, we did not show real (original) artworks but representations of artworks. This difference may have an effect on the hedonic ratings (Brieber, Leder, & Nadal, 2015). Furthermore, the JenAesthetics database consists of a preselected group of high-quality oil paintings. Hence, the database includes a large proportion of images of rather similar quality. Any differences in aesthetic ratings of these images may be relatively small, and therefore the aesthetic ratings may be rather stable across art styles and content matter. In addition, the analysis of ratings on *aesthetics* and *beauty* strongly depends from a proper understanding of these terms by the participants. If participants understood the terms wrongly, the conclusions drawn would be impaired.

## Conclusion

The analysis did not reveal evidence for universal image properties that are systematically linked to a higher aesthetic value in our sample of high-quality paintings. Instead, paintings from every art period show specific patterns of SIPs. As an exception, art portraits possess similar values of Self-Similarity over art periods. In an analysis of subjective rating scores, we found differences of ratings on artistic value (*aesthetics*) and individual liking (*beauty*). These differences in ratings were linked to SIPs, to the art period and to the time of origin of the paintings. Last but not least, we showed that groups of participants varied systematically in their hedonic preferences.

In summary, our study provides evidence that, to some extent, SIPs vary between art periods and subject matters and, in addition, they can be correlated with the subjective evaluation of paintings in a majority of the participants.

## Supplementary Material

Supplementary material

Supplementary material

Supplementary material

Supplementary material

Supplementary material

Supplementary material

Supplementary material

Supplementary material
