# Supplementary material for: Subjective Ratings of Beauty and Aesthetics: Correlations With Statistical Image Properties in Western Oil Paintings
Source: Iperception. 2017 Jun 28;8(3):2041669517715474. doi: 10.1177/2041669517715474 (PMC5496686; doi:10.1177/2041669517715474)
Supplement: Supplementary material [file supplementary_table1.pdf]

	Complexity	Anisotropy	Rule of Thirds	Color Hue	Color Saturation	Color Value	Aspect Ratio
Self-Similarity	**.358****	**-.483****	**-.108****	-.030	**-.145****	**.272****	**-.128****
Complexity	1	**-.349****	**-.167****	**.131****	**-.081****	**.244****	-.040
Anisotropy		1	**-.052***	**.083****	**-.051***	**.120****	**-.060***
Rule of Thirds			1	-.014	**.111****	**-.407****	**.138****
Color Hue				1	**-.349****	**.135****	.001
Color Saturation					1	**-.374****	**.126****
Color Value						1	**-.264****
