# Supplementary material for: Subjective Ratings of Beauty and Aesthetics: Correlations With Statistical Image Properties in Western Oil Paintings
Source: Iperception. 2017 Jun 28;8(3):2041669517715474. doi: 10.1177/2041669517715474 (PMC5496686; doi:10.1177/2041669517715474)
Supplement: Supplementary material [file supplementary_table2.pdf]

		Renaissance n = 110	Mannerism n = 36	Baroque n = 503	Rococo n = 93	Classicism n = 70	Romanticism n = 142	Realism n = 178	Impressionism n = 206	Symbolism n = 42	Post-Impressionism n = 202	Expressionism n = 32
Self-Similarity	M	0.853	0.854	0.862	0.862	0.847	0.878	0.871	0.882	0.870	0.888	0.875
	SD	0.049	0.040	0.049	0.046	0.053	0.046	0.043	0.033	0.056	0.036	0.025
Complexity	M	7.404	10.666	7.311	6.507	5.128	6.990	7.131	9.460	8.665	9.708	10.581
	SD	3.354	5.618	3.769	4.348	2.399	3.309	3.521	4.550	4.173	4.631	5.880
Anisotropy	M	0.000143	0.000126	0.000144	0.000173	0.000171	0.000159	0.000149	0.000152	0.000147	0.000154	0.000174
	SD	0.000056	0.000034	0.000061	0.000073	0.000079	0.000056	0.000053	0.000051	0.000046	0.000056	0.000071
Aspect Ratio	M	1.131	1.274	1.063	1.003	1.074	0.896	0.887	0.995	1.155	0.981	1.090
	SD	0.300	0.276	0.318	0.312	0.305	0.297	0.286	0.314	0.509	0.299	0.305
Rule of Thirds	M	0.211	0.209	0.224	0.218	0.225	0.221	0.221	0.202	0.224	0.195	0.191
	SD	0.047	0.039	0.060	0.055	0.044	0.065	0.051	0.045	0.064	0.033	0.039
Color Hue	M	0.202	0.242	0.203	0.195	0.193	0.196	0.213	0.272	0.229	0.289	0.317
	SD	0.092	0.109	0.115	0.122	0.108	0.120	0.120	0.124	0.132	0.106	0.100
Color Saturation	M	0.450	0.405	0.412	0.390	0.434	0.367	0.371	0.293	0.375	0.327	0.420
	SD	0.106	0.125	0.134	0.133	0.112	0.153	0.145	0.126	0.165	0.115	0.134
Color Value	M	0.356	0.321	0.341	0.391	0.345	0.400	0.425	0.518	0.457	0.507	0.496
	SD	0.089	0.104	0.127	0.121	0.088	0.132	0.132	0.128	0.141	0.126	0.114
