# Supplementary material for: Subjective Ratings of Beauty and Aesthetics: Correlations With Statistical Image Properties in Western Oil Paintings
Source: Iperception. 2017 Jun 28;8(3):2041669517715474. doi: 10.1177/2041669517715474 (PMC5496686; doi:10.1177/2041669517715474)
Supplement: Supplementary material [file supplementary_table3.pdf]

	aesthetics: regression coefficients B							
	Self-Similarity	Complexity	Anisotropy	Rule of Thirds	Color Hue	Color Saturation	Color Value	Aspect Ratio
Renaissance	0.195	-0.016	-0.026	-0.029	-0.056*	-0.048	-0.023	-0.041
Mannerism	0.108	-0.086*	<.001	-0.041	-0.073	-0.043	-0.029	-0.05
Baroque	0.064	-0.027	-0.012	-0.002	-0.038*	0.01	-0.022	0.023
Rococo	0.404	-0.036	-0.019	0.147*	-0.047*	0.085*	-0.133**	0.083
Classicism	-0.116	0.003	0.071	-0.01	0.001	0.019	0.066	-0.013
Romanticism	0.376	-0.007	0.002	0.001	-0.011	-0.014	0.047	-0.029
Realism	0.329	-0.008	-0.005	-0.023	-0.015	0.001	0.022	-0.048
Symbolism	0.031	-0.039	0.001	-0.058	0.001	-0.035	0.13*	-0.044
Post-Impressionism	-0.085	-0.139**	-0.069*	-0.046	-0.06*	-0.068*	-0.055	-0.089*
Expressionism	0.305	-0.142**	-0.173**	-0.204*	-0.152*	-0.077	0.031	-0.257**
Interaction Renaissance*respective SIP	-0.027	<.001	-4.467	-0.002	0.011	0.002	0.002	0.001
Interaction Mannerism*respective SIP	-0.02	<.001	-46.264	-0.01	0.004	-0.006	-0.003	<.001
Interaction Baroque*respective SIP	-0.01	<.001	-6.626	-0.009	0.007	-0.009	0.006	-0.004
Interaction Rococo*respective SIP	-0.048	<.001	1.596	-0.072**	0.018*	-0.026*	0.035*	-0.009*
Interaction Classicism*respective SIP	0.015	<.001	-36.01	0.011	0.006	-0.003	-0.01	0.003
Interaction Romanticism*respective SIP	-0.043	<.001	-6.398	-0.003	0.002	0.001	-0.01	0.002
Interaction Realism*respective SIP	-0.038	<.001	1.034	0.009	0.005	-0.002	-0.004	0.005
Interaction Symbolism*respective SIP	-0.007	<.001	-19.434	0.014	-0.012	0.001	-0.033*	0.002
Interaction Post-Impressionism*respective SIP	0	0.001*	-10.341	-0.02	-0.008	-0.005	-0.006	<.001
Interaction Expressionism*respective SIP	-0.053	<.001	8.278	0.026	-0.001	-0.019	-0.037*	0.01

	beauty: regression coefficients B							
	Self-Similarity	Complexity	Anisotropy	Rule of Thirds	Color Hue	Color Saturation	Color Value	Aspect Ratio
Renaissance	0.141	-0.094*	-0.063	-0.104	-0.089*	-0.136*	-0.048	-0.086
Mannerism	0.548	-0.138*	-0.063	-0.147	-0.121*	-0.142*	-0.005	-0.147
Baroque	0.217	-0.073**	-0.066*	-0.049	-0.065*	-0.028	-0.057	-0.004
Rococo	0.498	-0.069*	-0.061	0.122*	-0.072*	0.105*	-0.169**	0.08
Classicism	-0.06	-0.062	0.045	-0.036	-0.014	-0.025	0.03	-0.064
Romanticism	0.526	-0.001	-0.027	0.006	0.006	-0.003	0.055	0.003
Realism	0.718*	-0.035	-0.031	-0.054	-0.024	-0.036	0.022	-0.054
Symbolism	0.104	-0.063	-0.063	-0.065	0.004	-0.096*	0.131	-0.064
Post-Impressionism	0.027	-0.16**	-0.08*	-0.054	-0.053	-0.082*	-0.039	-0.081*
Expressionism	0.722	-0.184**	-0.205**	-0.243*	-0.164*	-0.143*	0.072	-0.267**
Interaction Renaissance*respective SIP	-0.027	-0.00009339	-23.053	0.003	-0.002	0.009	-0.005	<.001
Interaction Mannerism*respective SIP	-0.079	0.00007651	-49.814	0.008	-0.004	0.003	-0.027	0.002
Interaction Baroque*respective SIP	-0.033	0.00001281	-0.919	-0.008	0.001	-0.01	0.007	-0.006*
Interaction Rococo*respective SIP	-0.062	<.001	7.336	-0.075*	0.018	-0.038**	0.039**	-0.012*
Interaction Classicism*respective SIP	0.005	0.001	-46.831*	0.005	-0.005	-0.001	-0.007	0.003
Interaction Romanticism*respective SIP	-0.061	<.001	8.934	-0.006	-0.006	-0.002	-0.011	-0.002
Interaction Realism*respective SIP	-0.084*	<.001	9.261	0.018	0.005	0.005	-0.005	0.003
Interaction Symbolism*respective SIP	-0.017	<.001	8.813	0.008	-0.022	0.012	-0.037*	0.002
Interaction Post-Impressionism*respective SIP	-0.013	0.001*	-6.339	-0.019	-0.013	-0.002	-0.009	-0.001
Interaction Expressionism*respective SIP	-0.102	0.00008825	13.355	0.034	-0.005	-0.007	-0.049*	0.009
