# Supplementary material for: Subjective Ratings of Beauty and Aesthetics: Correlations With Statistical Image Properties in Western Oil Paintings
Source: Iperception. 2017 Jun 28;8(3):2041669517715474. doi: 10.1177/2041669517715474 (PMC5496686; doi:10.1177/2041669517715474)
Supplement: Supplementary material [file supplementary_table4.pdf]

	Self-Similarity		Complexity		Anisotropy		Aspect Ratio		Rule of Thirds		Color Hue		Color Saturation		Color Value	
	Landscape	Portrait (one Person)	Landscape	Portrait (one Person)	Landscape	Portrait (one Person)	Landscape	Portrait (one Person)	Landscape	Portrait (one Person)	Landscape	Portrait (one Person)	Landscape	Portrait (one Person)	Landscape	Portrait (one Person)
Renaissance	0.832	0.845	5.126	7.371	0.000190	0.000145	0.712	1.301	0.204	0.228	0.198	0.201	0.282	0.429	0.497	0.310
Mannerism	---	0.854	---	10.714	---	0.000124	---	1.335	---	0.212	---	0.232	---	0.381	---	0.283
Baroque	0.883	0.855	6.980	7.206	0.000152	0.000142	0.774	1.308	0.185	0.248	0.220	0.198	0.326	0.418	0.448	0.287
Rococo	0.878	0.851	5.611	4.719	0.000155	0.000196	0.743	1.279	0.208	0.232	0.184	0.189	0.431	0.379	0.386	0.311
Classicism	0.863	0.848	7.386	4.826	0.000092	0.000175	0.779	1.274	0.347	0.238	0.136	0.224	0.534	0.442	0.230	0.317
Romanticism	0.884	0.860	6.702	6.805	0.000164	0.000145	0.792	1.294	0.205	0.252	0.159	0.254	0.344	0.475	0.421	0.308
Realism	0.860	0.860	7.248	6.361	0.000147	0.000157	0.765	1.135	0.217	0.223	0.199	0.211	0.364	0.387	0.455	0.373
Impressionism	0.893	0.875	11.137	9.631	0.000147	0.000145	0.814	1.259	0.192	0.206	0.280	0.257	0.268	0.320	0.564	0.474
Symbolism	0.902	0.875	13.886	11.194	0.000171	0.000141	0.600	1.351	0.220	0.248	0.116	0.217	0.344	0.408	0.429	0.470
Post-Impressionism	0.902	0.877	10.960	8.368	0.000161	0.000150	0.869	1.294	0.185	0.205	0.292	0.279	0.311	0.339	0.529	0.446
Expressionism	0.861	0.872	21.293	9.978	0.000220	0.000171	0.973	1.238	0.230	0.201	0.277	0.341	0.388	0.452	0.537	0.486
All Periods	0.885	0.859	8.582	7.413	0.000156	0.000151	0.802	1.279	0.198	0.232	0.223	0.221	0.328	0.401	0.475	0.343
