# Supplementary material for: Subjective Ratings of Beauty and Aesthetics: Correlations With Statistical Image Properties in Western Oil Paintings
Source: Iperception. 2017 Jun 28;8(3):2041669517715474. doi: 10.1177/2041669517715474 (PMC5496686; doi:10.1177/2041669517715474)
Supplement: Supplementary material [file supplementary_table5.pdf]

	aesthetics: regression coefficient B							
	Self-Similarity	Complexity	Anisotropy	Rule of Thirds	Color Hue	Color Saturation	Color Value	Aspect Ratio
Abstract	.196	.026	-0.09	0.292	-0.023	0.197	-0.304	-0.099
Nearly abstract	1.042	.007	-0.15	-0.616	-0.086	0.636	0.338	-0.218
Landscapes	.344	.046*	0.078**	0.105*	0.071**	0.049*	0.035	0.037
Scenes with person(s)	-.018	.018	0.033*	0.045	0.028*	0.061*	0.029	-0.021
Still life	-.067	-.004	0.051	0.094*	0.065*	-0.027	0.061	-0.073
Flowers or vegetation	.492	.001	0.1*	<.001	0.012	-0.007	0.024	-0.071
Animals	.233	.020	-0.094	0.009	0.006	-0.003	-0.086	0.058
Seascape, port or coast	.137	.092**	0.111**	0.101*	0.112**	0.104**	0.09*	0.096
Sky	.946	.056	-0.158*	0.04	-0.094	0.073	-0.348*	-0.064
Portrait (many persons)	.004	.043	-0.002	0.046	0.037	0.053	0.046	-0.029
Nudes	.193	.036	-0.001	-0.058	0.042	-0.042	0.01	-0.048
Urban scene	.669	.074*	-0.021	0.01	0.032	0.128*	0.006	0.047
Building	1.007*	.094*	0.029	-0.024	0.158**	-0.024	0.233**	0.094
Interior scene	.198	.110	-0.047	0.46	0.103*	0.076	-0.206*	-0.102
Interaction Abstract*respective subject matter	-.032	-.001	5.496	-0.214	-0.016	-0.065	0.048	0.001
Interaction Nearly abstract*respective subject matter	-.123	-.001	61.052	0.338	0.007	-0.188	-0.06	0.017
Interaction Landscapes*respective subject matter	-.031	<.001	-10.136	-0.02	-0.004	0.005	0.006	0.001
Interaction Scenes with person(s)*respective subject matter	.005	<.001	-5.447	-0.009	-0.002	-0.009*	-0.001	0.004
Interaction Still life*respective subject matter	.011	.000	-19.602	-0.031	-0.017	0.012	-0.009	0.009
Interaction Flowers or vegetation*respective subject matter	-.053	.000	-53.867*	0.01	0.001	0.008	-0.001	0.008
Interaction Animals*respective subject matter	-.028	-.001	53.64	-0.012	-0.013	-0.004	0.017	-0.011
Interaction Seascape, port or coast*respective subject matter	-.005	<.001	-11.69	-0.004	-0.007	-0.002	0.001	-0.003
Interaction Sky*respective subject matter	-.111	-.002	59.299	-0.039	0.018	-0.065	0.054	<.001
Interaction Portrait (many persons)*respective subject matter	.005	<.001	33.694	<.001	0.004	-0.002	<.001	0.006
Interaction Nudes*respective subject matter	-.021	.000	6.285	0.034	-0.014	0.013	<.001	0.005
Interaction Urban scene*respective subject matter	-.069	.000	50.041	0.027	0.013	-0.02*	0.011	<.001
Interaction Building*respective subject matter	-.106*	.000	21.274	0.043	-0.038*	0.028*	-0.033*	-0.005
Interaction Interior scene*respective subject matter	-.015	-.001	63.661	-0.215*	-0.019	-0.002	0.061*	0.016

	beauty: regression coefficient B							
	Self-Similarity	Complexity	Anisotropy	Rule of Thirds	Color Hue	Color Saturation	Color Value	Aspect Ratio
Abstract	2.144	.116	-0.211	0.689	0.163	0.221	-0.117	-0.29
Nearly abstract	-.104	.066	0.078	-0.589	-0.025	0.76	0.264	-0.146
Landscapes	.435	.093**	0.148**	0.159**	0.121**	0.08*	0.132**	0.052
Scenes with person(s)	0.07	0.02	0.032	0.022	0.038*	0.056*	0.055*	-0.049
Still life	0.043	0.007	0.066	0.114*	0.078*	-0.041	0.098*	-0.138*
Flowers or vegetation	1.155*	0.092*	0.152*	0.102	0.026	0.078	0.079	-0.027
Animals	0.095	0.067	-0.056	0.064	0.042	0.047	-0.025	0.024
Seascape, port or coast	0.424	0.171**	0.175**	0.154*	0.177**	0.136**	0.213**	0.106
Sky	1.757*	0.2*	-0.224*	0.087	-0.104	0.171	-0.355*	-0.151
Portrait (many persons)	-0.123	0.016	-0.021	0.037	0.05	-0.014	0.027	-0.085
Nudes	-0.003	0.041	0.008	-0.065	0.077*	-0.067	-0.02	-0.106
Urban scene	0.495	0.136**	0.071	0.038	0.09*	0.178**	0.09	0.052
Building	1.517**	0.119*	0.112*	-0.069	0.207**	0.02	0.37**	0.134*
Interior scene	0.062	0.089	0.025	0.385*	0.092	0.111	-0.154	-0.132
Interaction Abstract*respective subject matter	-0.26	-0.002	70.555	-0.444	-0.07	-0.068	0.009	0.02
Interaction Nearly abstract*respective subject matter	0.012	-0.001	-52.584	0.355	0.006	-0.206*	-0.045	0.014
Interaction Landscapes*respective subject matter	-0.035	<.001	-19.305	-0.021	-0.001	0.012	-0.006	0.004
Interaction Scenes with person(s)*respective subject matter	-0.005	<.001	-1.607	0.003	-0.004	-0.006	-0.008	0.006*
Interaction Still life*respective subject matter	<.001	<.001	-16.344	-0.032	-0.016	0.02*	-0.016	0.016*
Interaction Flowers or vegetation*respective subject matter	-0.12*	<.001	-42.089	-0.009	0.022	0.002	-0.001	0.009
Interaction Animals*respective subject matter	-0.009	-0.001	52.148	-0.022	-0.013	-0.008	0.009	-0.004
Interaction Seascape, port or coast*respective subject matter	-0.03	<.001	-9.457	0.003	-0.007	0.009	-0.013	0.002
Interaction Sky*respective subject matter	-0.198*	-0.004*	116.448*	-0.042	0.037	-0.106*	0.058	0.014
Interaction Portrait (many persons)*respective subject matter	0.019	<.001	43.138	0.002	-0.004	0.013	0.003	0.01*
Interaction Nudes*respective subject matter	0.002	<.001	3.516	0.039	-0.027*	0.02	0.006	0.01*
Interaction Urban scene*respective subject matter	-0.042	<.001	33.027	0.045	0.016	-0.017	0.004	0.005
Interaction Building*respective subject matter	-0.156*	<.001	9.177	0.097	-0.032	0.034*	-0.051**	-0.004
Interaction Interior scene*respective subject matter	0.003	<.001	33.378	-0.165*	-0.002	-0.006	0.052	0.02*
