# Supplementary material for: Subjective Ratings of Beauty and Aesthetics: Correlations With Statistical Image Properties in Western Oil Paintings
Source: Iperception. 2017 Jun 28;8(3):2041669517715474. doi: 10.1177/2041669517715474 (PMC5496686; doi:10.1177/2041669517715474)
Supplement: Supplementary material [file supplementary_table6.pdf]

		Abstract n = 3	Nearly Abstract n = 7	Landscapes n = 178	Scenes with Person(s) n = 488	Still Life n = 66	Flowers or Vegetation n = 43	Animals n = 35	Seascape, Port or Coast n = 96
Self-Similarity	M	0.850	0.893	0.885	0.867	0.868	0.889	0.874	0.877
	SD	0.021	0.011	0.034	0.045	0.042	0.032	0.038	0.048
Complexity	M	10.535	9.215	8.582	7.572	8.706	12.051	7.150	7.831
	SD	2.979	5.816	4.993	4.016	4.293	5.868	3.067	4.131
Anisotropy	M	0.000200	0.000146	0.000156	0.000143	0.000137	0.000156	0.000144	0.000191
	SD	0.000069	0.000030	0.000055	0.000054	0.000053	0.000055	0.000040	0.000075
Aspect Ratio	M	1.046	0.833	0.802	0.975	0.975	0.998	0.853	0.736
	SD	0.325	0.286	0.196	0.312	0.267	0.286	0.271	0.145
Rule of Thirds	M	0.170	0.166	0.198	0.213	0.225	0.183	0.213	0.207
	SD	0.014	0.013	0.044	0.052	0.065	0.031	0.045	0.054
Color Hue	M	0.331	0.340	0.223	0.213	0.242	0.268	0.176	0.286
	SD	0.093	0.058	0.103	0.115	0.134	0.107	0.081	0.134
Color Saturation	M	0.421	0.369	0.328	0.414	0.410	0.331	0.355	0.261
	SD	0.061	0.043	0.121	0.134	0.153	0.112	0.130	0.123
Color Value	M	0.474	0.659	0.475	0.382	0.396	0.468	0.418	0.547
	SD	0.070	0.068	0.122	0.118	0.161	0.137	0.143	0.143

		Sky n = 11	Portrait (one Person) n = 439	Portrait (many Persons) n = 79	Nudes n = 42	Urban Scenes n = 65	Buildings n = 36	Interior Scenes n = 14	Other Subject Matters n = 12
Self-Similarity	M	0.889	0.859	0.866	0.862	0.880	0.888	0.870	0.854
	SD	0.047	0.052	0.043	0.049	0.036	0.035	0.043	0.056
Complexity	M	5.798	7.413	7.190	7.087	8.727	9.505	7.671	8.326
	SD	2.498	3.866	3.229	3.612	4.696	3.833	2.232	3.728
Anisotropy	M	0.000182	0.000151	0.000144	0.000154	0.000162	0.000145	0.000176	0.000155
	SD	0.000082	0.000064	0.000050	0.000058	0.000055	0.000055	0.000069	0.000043
Aspect Ratio	M	0.745	1.279	1.083	1.050	0.793	0.900	0.994	1.029
	SD	0.125	0.232	0.283	0.435	0.209	0.322	0.304	0.509
Rule of Thirds	M	0.230	0.232	0.224	0.201	0.195	0.199	0.181	0.208
	SD	0.081	0.059	0.045	0.041	0.043	0.032	0.036	0.037
Color Hue	M	0.256	0.221	0.218	0.237	0.223	0.262	0.197	0.215
	SD	0.155	0.125	0.121	0.130	0.118	0.115	0.132	0.092
Color Saturation	M	0.171	0.401	0.412	0.398	0.315	0.308	0.375	0.371
	SD	0.069	0.136	0.136	0.125	0.119	0.129	0.137	0.118
Color Value	M	0.565	0.343	0.380	0.439	0.512	0.517	0.446	0.424
	SD	0.111	0.129	0.119	0.122	0.124	0.128	0.114	0.140
